# Editorial: Women in oral microbes and host: 2025

**DOI:** 10.3389/fcimb.2026.1968328

**Published:** 2026-09-08

**Authors:** Catherine A. Butler, Angela C. Brown

**Affiliations:** 1 Melbourne Dental School, The University of Melbourne, Melbourne, VIC, Australia; 2 Department of Chemical and Biomolecular Engineering, Bethlehem, PA, United States

**Keywords:** dysbiosis, host-microbe, microbial community, oral microbiome, periodontal disease

The field of oral microbiology has undergone a paradigm shift, moving beyond the localized study of dental decay toward a comprehensive understanding of the oral-systemic axis. This evolution is particularly evident in this Research Topic, *“Women in Oral Microbes and Host: 2025,”* a theme that emphasizes the unique biological intersections between the host immune response and the complex oral microbiome. Crucially, this collection of articles is authored and led by female scientists, highlighting the significant contributions of women researchers in unraveling the complexities of the oral microbiome.

The foundation of lifelong oral health is established in early life and is heavily influenced by maternal factors. Arishi et al. provide a critical examination of this dynamic in their study of mother-infant dyads, revealing that concentrations of key components present in human milk directly influence the composition and diversity of the infant oral microbiome. Interestingly, their findings suggest that milk components, such as antimicrobial proteins (AMPs) and minerals, act more like a “mouth wash” than a “medicine,” implying that local, direct mechanisms exert a stronger influence on the assembly of the infant oral microbiome than cumulative systemic effects due to intake. This work underscores the maternal role in guiding early microbial colonization, highlighting human milk as a complex biofluid that provides both nutrition and direct ecological regulation.

While fungi account for only a small proportion of the microbiome in terms of biomass, they can exert a disproportionately large influence on microbial community dynamics and oral health. In their comprehensive review of the pediatric oral mycobiome, Xiang and Liu note that the oral cavity hosts a diverse mycobiome of approximately 100 species, with *Candida* being the most prevalent genus. Recent advances in sequencing technologies have enhanced our understanding of the role of the mycobiome in oral health. By emphasizing inter-kingdom interactions, such as the synergy between *Candida albicans* and *Streptococcus mutans*, the authors demonstrate how the fungal components of the microbiome shape immune development and influence susceptibility to oral diseases such as dental caries.

The utility of the oral microbiome as a diagnostic window is explored by Tu et al., who investigate the relationship between tongue-coating microbiota and non-puerperal mastitis (NPM), an inflammatory breast disease in women. To improve on current methods to diagnose NPM, this study integrates AI-quantified tongue imaging with 16S rRNA gene sequencing to build a non-invasive diagnostic framework. Their Gradient Boosting Decision Tree (GBDT) model achieved a remarkable diagnostic accuracy of 0.95, identifying *Campylobacter* and *Alloprevotella* as key microbial predictors of NPM. This research provides scientific evidence for the traditional practice of tongue diagnosis and demonstrates that the oral microbiome is a sensitive indicator of systemic pathological changes in women.

Ryan et al. demonstrate that the composition of the oral microbiome is also influenced by host physiology through their longitudinal study on vitamin D deficiency. Using a murine crossover model, they show that dietary vitamin D deficiency affects microbial community development, promoting a dysbiotic microbiome associated with inflammation and alveolar bone loss. Single-cell RNA sequencing of gingival and buccal tissues revealed that vitamin D deficiency was accompanied by a reduction in epithelial cell populations and an increased expression of inflammatory genes. Collectively, their results identify vitamin D as an important modulator of host-microbe interactions within the oral cavity and suggest that maintaining adequate vitamin D levels may help preserve oral health, even in the presence of microbial dysbiosis.

In addition to host systemic factors, the specific regulatory mechanisms within pathogens themselves determine their ability to survive within the host environment. Yanamandra et al. characterize RNA-binding protein *Porphyromonas gingivalis* 1 (RbpPg1), the first RNA-binding protein identified in the periodontal pathogen *P. gingivalis*. Their research reveals that RbpPg1 is essential for bacterial survival within host cells and adaption to environmental stress, particularly zinc toxicity. By binding to the transcript of a zinc efflux pump, RbpPg1 modulates intracellular metal levels and protease activity, which are critical for the pathogen’s virulence. This study provides a molecular-level understanding of how oral pathogens navigate the hostile environment of the human host, using unique RNA-binding motifs to fine-tune their survival strategies.

The composition and function of the oral microbiome are influenced not only by host physiology but also by external environmental factors, including the increasing use of electronic cigarettes. Khodabakhsh Majd et al. investigate how E-cigarette liquids (E-liquids) alter the biogenesis of outer membrane vesicles (OMVs) by the oral pathogen, *Aggregatibacter actinomycetemcomitans*. While E-liquids inhibited bacterial growth at high concentrations, subinhibitory exposure shifted OMV production toward larger vesicles with increased protein and altered protein composition. Since OMVs play important roles in the delivery of toxins such as leukotoxin A to host cells, these findings suggest that vaping may enhance the pathogenicity of periodontal bacteria. This work adds a contemporary dimension to the topic, highlighting how lifestyle choices directly modulate the virulence mechanisms of the oral microbiome.

Finally, the Research Topic concludes with a forward-looking perspective on oral health management. Katrak et al. provide a mini-review of oral hygiene agents, focusing on their biochemical and ecological effects on *S. mutans*. They advocate for combinatorial and synergistic therapies, such as pairing fluoride with prebiotics like arginine or xylitol. This ecological approach to oral health management aligns with the broader themes of the other articles, emphasizing the need to move beyond simple antimicrobial strategies toward the restoration of microbial homeostasis within the host.

Together, the articles in this Research Topic highlight the complexity of host-microbe interactions within the oral cavity and their implications for human health. Advances in high-resolution sequencing, multi-omics approaches, and computational analyses are providing unprecedented insight into the mechanisms governing these interactions. As our understanding of the oral microbiome continues to deepen, these discoveries will drive the development of more precise diagnostics, targeted therapeutics, and personalized strategies for disease prevention and treatment. Together, these contributions highlight both the progress made and the exciting opportunities that lie ahead for improving oral and systemic health through a more comprehensive understanding of the host–microbiome interface.

